# Hepatoprotective effects of sevoflurane against hepatic ischemia-reperfusion injury by regulating microRNA-124-3p-mediated TRAF3/CREB axis

**DOI:** 10.1038/s41420-021-00784-7

**Published:** 2022-03-08

**Authors:** Yi-Liang Wang, Ying Zhang, Da-Sheng Cai

**Affiliations:** 1 Department of Anaesthesiology, The First Hospital of China Medical University, Shenyang, 110001 PR China; 2 Department of Thyroid and Breast Surgery, Liaoning Provincial People’s Hospital, Shenyang, 110001 PR China

**Keywords:** Cell biology, Diseases

## Abstract

The purpose of the present study is to define the role of sevoflurane (SEV) in hepatic ischemia-reperfusion (I/R) injury as well as its underlying mechanism. Initially, hepatic I/R animal models and I/R hepatocyte models were established in C57BL/6 mice and normal mouse hepatocytes (BNL CL.2) after SEV preconditioning, respectively, followed by detection of microRNA-124-3p (miR-124-3p), TRAF3, and CREB expression by RT-qPCR and Western blot analysis. In addition, miR-124-3p, TRAF3 and CREB expression in hepatocytes was altered to identify their roles in modulating the levels of glutathione transferase (GST), aspartate aminotransferase (AST) and alanine aminotransferase (ALT), and inflammation-related factors and hepatocyte apoptosis by ELISA and flow cytometry respectively. The effects of SEV on the miR-124-3p/TRAF3/CREB axis were also verified in vitro and in vivo. IP assay was performed to verify the effect of TRAF3 on CREB ubiquitination in BNL CL.2 cells, and the cycloheximide (CHX) intervention experiment to detect the stability of CREB protein. SEV augmented the miR-124-3p expression in I/R animal and cell models. Moreover, SEV was observed to suppress I/R-induced liver damage, GST, ALT, and AST levels, hepatocyte apoptosis and inflammation. Overexpression of miR-124-3p resulted in alleviation of hepatic I/R injury, which was countered by TRAF3 overexpression. miR-124-3p targeted TRAF3, while TRAF3 promoted CREB ubiquitination and reduced protein stability of CREB. SEV could impede I/R-induced liver damage, GST, ALT, and AST levels, hepatocyte apoptosis and inflammation via mediation of the miR-124-3p/TRAF3/CREB axis in vitro and in vivo. Collectively, SEV may upregulate miR-124-3p to inhibit TRAF3 expression, thereby reducing the ubiquitination and degradation of CREB, alleviating hepatic I/R injury.

## Introduction

Hepatic ischemia-reperfusion (I/R) injury is a common occurrence following blood supply restoration after blood vessel occlusion during the course of countless liver surgeries [1]. Furthermore, hepatic I/R injury is recognized as the leading cause of abnormal liver function and hepatic failure, whereas there are only a limited number of options to effectively tackle this major complication of liver surgery [2]. In addition, hepatic I/R injury has long been a major cause of liver dysfunction and failure after surgical procedures [3, 4]. Besides, hepatic I/R injury implicates a persistent pro-oxidant and pro-inflammatory mechanisms that result in hepatocyte dysfunction and inflammatory or apoptotic responses [5, 6]. Notably, pretreatment with sevoflurane (SEV) has been reported to protect liver tissues against I/R injury, as well as confer protection to hepatocytes against I/R-triggered necrosis [7]. However, the molecular mechanisms of SEV underlying the highly-beneficial hepatoprotection remain largely unknown.

Recently, studies have uncovered the association between microRNAs (miRNAs) and liver protection conferred by anesthetic preconditioning in hepatic I/R injury [8]. miRNAs are small non-coding RNAs endogenously expressed to serve the function of posttranscriptional gene expression mediation [9]. For example, miR-182-5p has been proposed to suppress liver damage and inflammation by targeting TLR4 in an animal model of hepatic IR injury and in a cell model of lipopolysaccharide-induced inflammation [10]. Interestingly, prior evidence has also suggested that miR-124-3p was downregulated in hepatic I/R injury, whereas its overexpression was closely-related to arrested inflammation in vivo and in vitro [11]. Furthermore, another study has indicated that the mechanism underlying the SEV hepatoprotection function may be associated with the upregulation of said miR-124-3p [12]. Initial bioinformatics analysis in the current study further identified the necrosis factor (TNF) receptor-associated factor 3 (TRAF3) gene as a putative target of miR-124-3p. TRAF3 has been known to be mediated by and contributes to multiple ubiquitination actions, and plays a wide range of regulatory roles in inflammatory reactions [13]. The evidence presented by Wang et al. demonstrated that TRAF3 was highly-expressed in human liver samples and animal liver with hepatic steatosis, and its interaction with TAK1 contributed to the modulation of ubiquitination in hepatocytes [14]. Furthermore, TRAF3 has also been noted to repress the stability of cAMP-responsive element-binding protein 3 (CREB3), as well as to augment the process of CREB ubiquitination [15]. Moreover, CREB upregulation has also been previously demonstrated to protect the brain from I/R injury in vivo [16]. Therefore, we hypothesized that miR-124-3p/TRAF3/CREB axis participates in the protective effects of SEV against hepatic IR injury.

## Results

### SEV promotes miR-124-3p expression to alleviate hepatic I/R injury

**Fig. 1:**
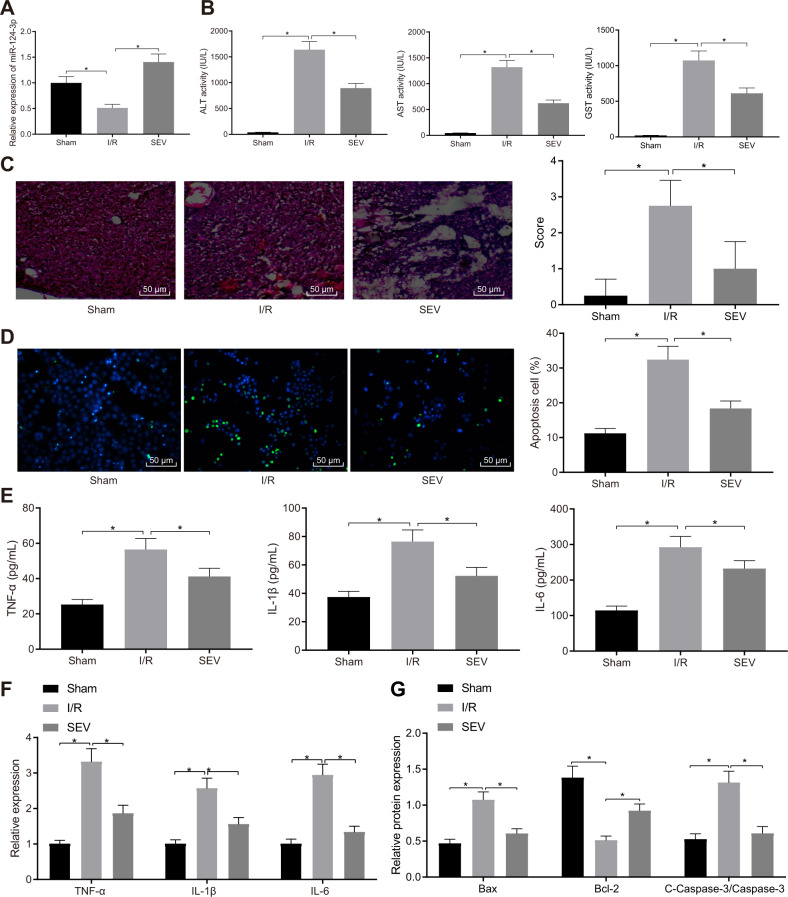
SEV promotes the expression of miR-124-3p and alleviates hepatic I/R injury in mice. **A** The expression of miR-124-3p in liver tissues of sham-operated mice, I/R mice or I/R mice treated with SEV detected by RT-qPCR. **B** Serum levels of GST, ALT, and AST in sham-operated mice, I/R mice or I/R mice treated with SEV detected by ELISA. **C** Liver tissue injury in sham-operated mice, I/R mice or I/R mice treated with SEV determined by HE staining (200×). **D** Hepatocyte apoptosis in sham-operated mice, I/R mice or I/R mice treated with SEV detected by TUNEL staining (200×). **E** Serum levels of TNF-α, IL-1β, and IL-6 in sham-operated mice, I/R mice or I/R mice treated with SEV detected by ELISA. **F** The expression of TNF-α, IL-1β, and IL-6 in liver tissues of sham-operated mice, I/R mice or I/R mice treated with SEV detected by RT-qPCR. **G** The protein expression of Bax, Bcl-2, and cleaved-caspase-3 in liver tissues of sham-operated mice, I/R mice or I/R mice treated with SEV detected by Western blot analysis. *p < 0.05 (*n* = 8).

It has been documented that SEV pretreatment could repress I/R injury by protecting live tissues and conferring protection to hepatocytes against I/R-triggered necrosis [7]. Moreover, the mechanism underlying the SEV hepatoprotection function may be associated with the upregulation of miR-124-3p [12]. In addition, miR-124-3p was downregulated in hepatic I/R injury [11]. Therefore, C57BL/6 mice were employed to establish hepatic I/R models after SEV intervention. Reverse transcription quantitative polymerase chain reaction (RT-qPCR) demonstrated that compared with sham-operated mice, I/R mice exhibited decreased miR-124-3p expression, whereas I/R mice treated with SEV presented with elevated miR-124-3p expression relative to I/R mice (Fig. 1A). Enzyme-linked immunosorbent assay (ELISA) showed that serum levels of glutathione transferase (GST), alanine aminotransferase (ALT), and aspartate aminotransferase (AST) were higher in I/R mice than in sham-operated mice, while further SEV treatment reduced GST, ALT, and AST levels significantly in the I/R mice, which suggested alleviated liver injury (Fig. 1B). As shown by hematoxylin and eosin (HE) staining and terminal deoxynucleotidyl transferase-mediated dutp-biotin nick end labeling (TUNEL) staining, I/R mice displayed more severe liver tissue injury and increased hepatocyte apoptosis compared to sham-operated mice, which was abrogated by SEV treatment (Fig. 1C, D).

Subsequently, ELISA and RT-qPCR results further demonstrated that necrosis factor-α (TNF-α), interleukin-1β (IL-1β), and IL-6 levels in serum and liver tissues of I/R mice were notably higher than that in sham-operated mice, whereas a significant reduction in the aforementioned indicators was observed in I/R mice treated with SEV compared to I/R mice (Fig. 1E, F). Moreover, Western blot analysis determined that relative to sham-operated mice, I/R mice displayed an increase in cleaved-caspase-3 and Bcl-2-associated X protein (Bax) expression as well as a decrease in B-cell lymphoma 2 (Bcl-2) expression, whereas all the said changes were reversed by SEV treatment (Fig. 1G and Supplementary Fig. 1A).

### SEV inhibits I/R Injury of hepatocytes via upregulation of miR-124-3p

To determine whether SEV could inhibit I/R injury of hepatocytes by promoting expression of miR-124-3p, I/R cell models were induced on BNL CL.2 (mouse fetal hepatocytes) using H_2_O_2_. miR-124-3p expression was increased with the increasing concentration of H_2_O_2_, and stabilized at the concentration of 200 μM (Supplementary Fig. 2A).

**Fig. 2:**
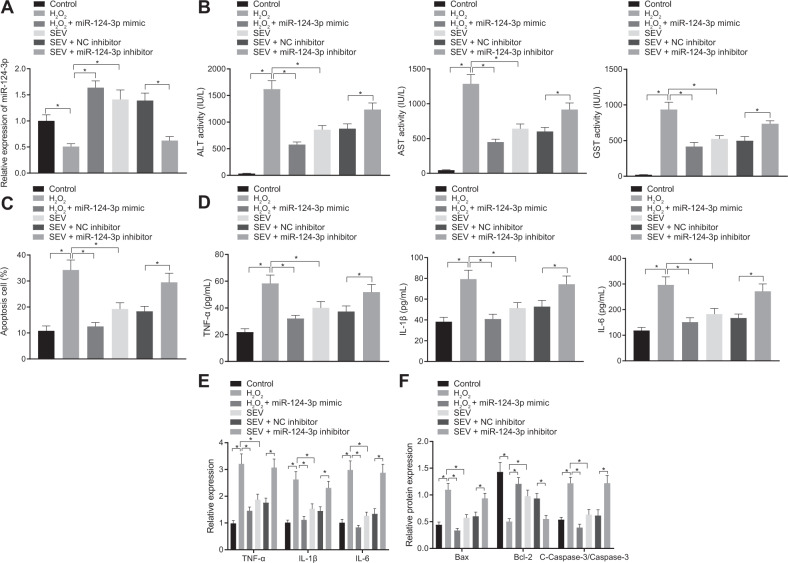
SEV inhibits the occurrence of I/R injury of hepatocytes by increasing the expression of miR-124-3p. **A** The expression of miR-124-3p in hepatocytes in the presence of SEV or SEV + miR-124-3p inhibitor detected by RT-qPCR. **B** Serum levels of GST, ALT, and AST in hepatocyte supernatant in the presence of SEV or SEV + miR-124-3p inhibitor detected by ELISA. **C** Hepatocyte apoptosis in the presence of SEV or SEV + miR-124-3p inhibitor detected by flow cytometry. **D** The expression of TNF-α, IL-1β, and IL-6 in hepatocyte supernatant in the presence of SEV or SEV + miR-124-3p inhibitor determined by ELISA. **E** The expression of TNF-α, IL-1β, and IL-6 in hepatocytes in the presence of SEV or SEV + miR-124-3p inhibitor detected by RT-qPCR. **F** The protein expression of Bax, Bcl-2, and cleaved-caspase-3 in the supernatant of hepatocytes in the presence of SEV or SEV + miR-124-3p inhibitor detected by Western blot analysis. *p < 0.05. The cell experiment was repeated three times.

RT-qPCR revealed that H_2_O_2_ treatment led to decreased miR-124-3p expression, which could be elevated by further treatment with SEV or miR-124-3p mimic. However, SEV + miR-124-3p inhibitor brought about a marked decline in miR-124-3p expression compared with SEV + NC inhibitor (Fig. 2A). In addition, ELISA showed that serum levels of GST, ALT, and AST were increased after H_2_O_2_ treatment, and could be reversed by further treatment with SEV or miR-124-3p mimic. Moreover, SEV + miR-124-3p inhibitor reversed the decreased serum levels of GST, ALT, and AST caused by treatment with SEV in H_2_O_2_-treated hepatocytes (Fig. 2B). Flow cytometry further demonstrated elevated apoptosis rate in hepatocytes solely subjected to H_2_O_2_ treatment, which was negated following additional treatment with SEV or miR-124-3p mimic. In contrast to treatment of SEV + NC inhibitor, H_2_O_2_-treated hepatocyte apoptosis was elevated in response to treatment with SEV + miR-124-3p inhibitor (Fig. 2C).

Moreover, ELISA and RT-qPCR results displayed that TNF-α, IL-1β, and IL-6 levels were all elevated in the supernatant of H_2_O_2_-treated hepatocytes and in H_2_O_2_-treated hepatocytes, which was counteracted by further treatment with SEV or miR-124-3p mimic. Meanwhile, in the presence of SEV, miR-124-3p inhibitor treatment enhanced TNF-α, IL-1β, and IL-6 levels in the supernatant of H_2_O_2_-treated hepatocytes and in H_2_O_2_-treated hepatocytes (Fig. 2D, E). Meanwhile, H_2_O_2_ treatment resulted in an increase in cleaved-caspase-3 and Bax expression, while decreasing that of Bcl-2, which was neutralized by further treatment with SEV or miR-124-3p mimic. SEV + miR-124-3p inhibitor further reversed changes caused by treatment with SEV in H_2_O_2_-treated hepatocytes (Fig. 2F).

### miR-124-3p targets and downregulates TRAF3, thus inhibiting H_2_O_2_-induced hepatocyte apoptosis and release of inflammatory factors

**Fig. 3:**
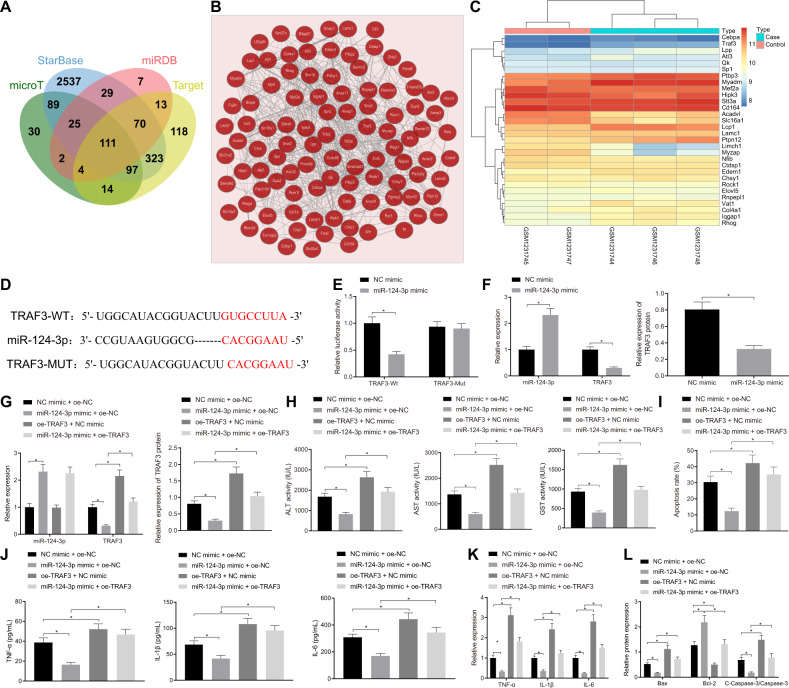
miR-124-3p downregulates the expression of TRAF3 and thus inhibits H_2_O_2_-induced hepatocyte apoptosis and release of inflammatory factors. **A** The Venn map for intersecting downstream target genes of miR-124-3p predicted on bioinformatics websites, microT, starBase, miRDB, and TargetScan. **B** The co-expression network of the 111 candidate target genes obtained from the Coexpedia website. **C** Differential expression of the 30 genes (with a high score in co-expression network) in the GSE50884 dataset. **D** The binding site between miR-124-3p and TRAF3 predicted by the starBase website. **E** The targeting relationship between miR-124-3p and TRAF3 verified by dual-luciferase reporter gene assay. **F** The expression of TRAF3 in the presence of miR-124-3p mimic detected by RT-qPCR and Western blot analysis. **G** The expression of miR-124-3p and TRAF3 in hepatocytes in the presence of miR-124-3p mimic, oe-TRAF3 or miR-124-3p mimic + oe-TRAF3 detected by RT-qPCR and Western blot analysis. **H** Serum levels of GST, ALT, and AST in hepatocyte supernatant in the presence of miR-124-3p mimic, oe-TRAF3 or miR-124-3p mimic + oe-TRAF3 detected by ELISA. **I** Hepatocyte apoptosis in the presence of miR-124-3p mimic, oe-TRAF3 or miR-124-3p mimic + oe-TRAF3 detected by flow cytometry. **J** The expression of TNF-α, IL-1β, and IL-6 in hepatocyte supernatant in the presence of miR-124-3p mimic, oe-TRAF3 or miR-124-3p mimic + oe-TRAF3 detected by ELISA. **K** The expression of TNF-α, IL-1β, and IL-6 in hepatocytes in the presence of miR-124-3p mimic, oe-TRAF3 or miR-124-3p mimic + oe-TRAF3 detected by RT-qPCR. **L** The protein expression of Bax, Bcl-2, and cleaved-caspase-3 in hepatocytes in the presence of miR-124-3p mimic, oe-TRAF3 or miR-124-3p mimic + oe-TRAF3 detected by Western blot analysis. *p < 0.05. The cell experiment was repeated three times.

The downstream target genes of miR-124-3p were predicted using online bioinformatics databases, microT, starBase, miRDB, and TargetScan. The prediction results reared a total of 372, 3281, 261, and 750 genes respectively, while 111 intersecting genes were obtained from these four prediction results (Fig. 3A). The co-expression network of the 111 candidate target genes was then obtained using the Coexpedia website (Fig. 3B), among which 30 genes exhibited a high score in this network (score >22). The differential expression of the aforementioned 30 genes in the GSE50884 dataset was then determined, and highly-expressed TRAF3 was found in I/R, with the most significant *p* value (Fig. 3C). The starBase website further predicted the presence of binding sites between miR-124-3p and TRAF3 (Fig. 3D), and dual-luciferase reporter gene assay demonstrated that miR-124-3p mimic failed to bring about significant change in the luciferase activity of mutant type (MUT) TRAF3, but notably diminished the luciferase activity of wild type (WT) TRAF3 (Fig. 3E). miR-124-3p mimic contributed to increased miR-124-3p expression, but decreased TRAF3 expression, indicating that miR-124-3p could target and inhibit the TRAF3 gene (Fig. 3F and Supplementary Fig. 1B).

Next, miR-124-3p mimic increased miR-124-3p expression but reduced that of TRAF3, whereas oe-TRAF3 brought about an increase in TRAF3 expression. However, treatment with miR-124-3p mimic + oe-TRAF3 resulted in a marked increase in TRAF3 expression in BNL CL.2 cells relative to the treatment of miR-124-3p mimic + oe-NC (Fig. 3G and Supplementary Fig. 1C). ELISA results illustrated decreases in GST, ALT, and AST serum levels in the supernatant of hepatocytes following treatment with miR-124-3p mimic, whereas increases in GST, ALT, and AST serum levels were observed after treatment with oe-TRAF3. Relative to treatment with miR-124-3p mimic + oe-NC, GST, ALT, and AST serum levels were found to be increased in treatment with miR-124-3p mimic + oe-TRAF3 (Fig. 3H). Meanwhile, flow cytometric data demonstrated that miR-124-3p mimic led to a decrease in hepatocyte apoptosis, but oe-TRAF3 showed an opposite trend, whereas miR-124-3p mimic + oe-TRAF3 led to increased hepatocyte apoptosis relative to miR-124-3p mimic + oe-NC (Fig. 3I). ELISA and RT-qPCR results demonstrated that TNF-α, IL-1β, and IL-6 levels were all declined in hepatocyte supernatant and hepatocytes upon miR-124-3p mimic, which was normalized by TRAF3 overexpression. Meanwhile, miR-124-3p mimic + oe-TRAF3 brought about increased TNF-α, IL-1β, and IL-6 levels in hepatocyte supernatant and hepatocytes relative to miR-124-3p mimic + oe-NC (Fig. 3J, K). Furthermore, Western blot analysis demonstrated that miR-124-3p mimic resulted in decreases in cleaved-caspase-3 and Bax in expression, as well as an increase in expression of Bcl-2, which could all be abolished by TRAF3 overexpression. Elevated cleaved-caspase-3 and Bax expression, as well as reduced expression of Bcl-2 was detected upon miR-124-3p mimic + oe-TRAF3 relative to miR-124-3p mimic + oe-NC (Fig. 3L and Supplementary Fig. 1D).

### TRAF3 negatively regulates CREB expression through ubiquitination, thus promoting H_2_O_2_-induced hepatocyte apoptosis and release of inflammatory factors

**Fig. 4:**
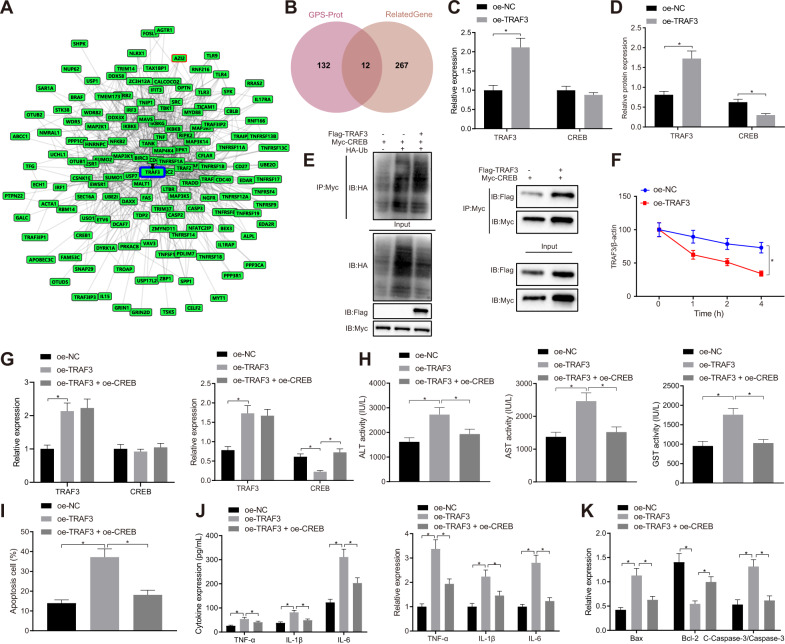
TRAF3 attenuates the expression of CREB through ubiquitination, thus promoting the H_2_O_2_-induced hepatocyte apoptosis and release of inflammatory factors. **A** The GPS-Prot interaction network of TRAF3 in the GeneCards database. **B** The Venn map of the 12 intersecting genes interacted with TRAF and related to hepatic I/R injury. **C** The mRNA expression of TRAF3 and CREB in the presence of oe-TRAF3 or oe-TRAF3 + oe-CREB detected by RT-qPCR. **D** The protein expression of TRAF3 and CREB in the presence of oe-TRAF3 or oe-TRAF3 + oe-CREB detected by Western blot analysis. **E** The effects of TRAF3 on CREB ubiquitination detected by IP assay. **F** The effects of TRAF3 on CREB protein stability after CHX intervention. **G** The expression of CREB and TRAF3 in hepatocytes in the presence of oe-TRAF3 or oe-TRAF3 + oe-CREB detected by RT-qPCR and Western blot analysis. **H** Serum levels of GST, ALT, and AST in hepatocyte supernatant in the presence of oe-TRAF3 or oe-TRAF3 + oe-CREB detected by ELISA. **I** Hepatocyte apoptosis in the presence of oe-TRAF3 or oe-TRAF3 + oe-CREB detected by flow cytometry. **J** The expression of TNF-α, IL-1β, and IL-6 in hepatocyte supernatant and hepatocytes in the presence of oe-TRAF3 or oe-TRAF3 + oe-CREB detected by ELISA and RT-qPCR. **K** The protein expression of Bax, Bcl-2, and cleaved-caspase-3 in hepatocytes in the presence of oe-TRAF3 or oe-TRAF3 + oe-CREB detected by Western blot analysis. *p < 0.05. The cell experiment was repeated three times.

In order to further explore the downstream regulatory factors of TRAF3, the GPS-Prot interaction network of TRAF3 (Fig. 4A) was retrieved using the GeneCards database. The results showed that there was a total of 279 hepatic I/R injury-related genes. Using the Jvenn tool, 12 intersecting genes were found interacting with TRAF and correlated with hepatic I/R injury (Fig. 4B).

TRAF3 has been exhibited to accelerate the process of CREB ubiquitination [15]. A prior study manifested the participation of CREB upregulation in repression of hepatic I/R injury [17]. Results showed that TRAF3 expression was notably increased, while the CREB protein expression was markedly decreased by oe-TRAF3, along with insignificantly changed CREB mRNA expression (Fig. 4C, D and Supplementary Fig. 1E). Myc-CREB, Myc-CREB + hemagglutinin (HA)-ubiquitin (Ub), or Myc-CREB + HA-Ub + Flag-TRAF3 were subsequently transfected into BNL CL.2 hepatocytes, and Myc-CREB + HA-Ub + Flag-TRAF3 was used in immunoprecipitation (IP) assay to detect the ubiquitination modification of CREB. Relative to Myc-CREB, Myc-CREB + HA-Ub resulted in upregulated ubiquitination level of CREB, which were further increased upon Myc-CREB + HA-Ub + Flag-TRAF3. Meanwhile, Myc-CREB or Myc-CREB + Flag-TRAF3 was transfected into BNL CL.2 hepatocytes, and the interaction between CREB and TRAF3 was detected with IP assay using Myc. TRAF3 bands were then detected in the presence of Myc-CREB + Flag-TRAF3, indicating that CREB could interact with TRAF3 (Fig. 4E). The results displayed that the degradation rate of CREB was increased following oe-TRAF3 treatment (Fig. 4F and Supplementary Fig. 1F).

Then, oe-TRAF3 led to increased expression of TRAF3, but reduced CREB protein expression in H_2_O_2_-treated BNL CL.2 cells, along with unchanged CREB mRNA expression. However, a combination of oe-TRAF3 and oe-CREB resulted in a marked elevation of CREB protein expression with no significant changes in TRAF3 expression and CREB mRNA expression when compared to the treatment with oe-TRAF3 (Fig. 4G and Supplementary Fig. 1G). In addition, ELISA demonstrated that GST, ALT, and AST serum levels were elevated by oe-TRAF3, and could be reversed by additional transfection with oe-CREB (Fig. 4H). Additionally, flow cytometry showed that oe-TRAF3 led to augmented hepatocyte apoptosis, which was rescued by additional treatment oe-CREB (Fig. 4I). ELISA and RT-qPCR results revealed that oe-TRAF3 resulted in elevated TNF-α, IL-1β, and IL-6 levels in hepatocyte supernatant and hepatocytes, which was neutralized by further CREB overexpression (Fig. 4J). Western blot analysis results displayed that oe-TRAF3 brought about an increase in cleaved-caspase-3 and Bax expression and decreased that of Bcl-2. However, these changes were reversed in entirety following oe-TRAF3 + oe-CREB treatment (Fig. 4K and Supplementary Fig. 1H).

### SEV orchestrates miR-124-3p/TRAF3/CREB axis to inhibit H_2_O_2_-induced hepatocyte apoptosis and release of inflammatory factors

**Fig. 5:**
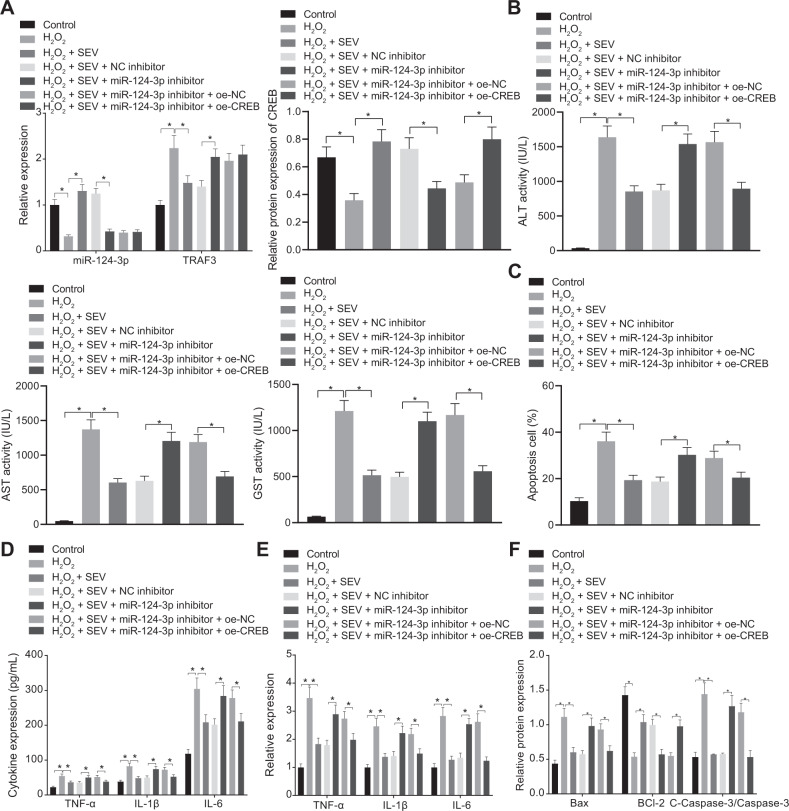
SEV inhibits H_2_O_2_-induced hepatocyte apoptosis and release of inflammatory factors through the miR-124-3p/TRAF3/CREB axis. **A** The expression of miR-124-3p and TRAF3 determined by RT-qPCR and CREB protein expression measured by Western blot analysis in hepatocytes in the presence of SEV, SEV + miR-124-3p inhibitor, or SEV + miR-124-3p inhibitor + oe-CREB. **B** Serum levels of GST, ALT, AST, and GST in hepatocyte supernatant in the presence of SEV, SEV + miR-124-3p inhibitor, or SEV + miR-124-3p inhibitor + oe-CREB detected by ELISA. **C** Hepatocyte apoptosis in the presence of SEV, SEV + miR-124-3p inhibitor, or SEV + miR-124-3p inhibitor + oe-CREB detected by flow cytometry. **D** The expression of TNF-α, IL-1β, and IL-6 in hepatocyte supernatant in the presence of SEV, SEV + miR-124-3p inhibitor, or SEV + miR-124-3p inhibitor + oe-CREB detected by ELISA. **E** The expression of TNF-α, IL-1β, and IL-6 in hepatocytes in the presence of SEV, SEV + miR-124-3p inhibitor, or SEV + miR-124-3p inhibitor + oe-CREB detected by RT-qPCR. **F** The protein expression of Bax, Bcl-2, and cleaved-caspase-3 in hepatocytes in the presence of SEV, SEV + miR-124-3p inhibitor or SEV + miR-124-3p inhibitor + oe-CREB detected by Western blot analysis. *p < 0.05. The cell experiment was repeated three times.

Results illustrated reduced miR-124-3p expression and CREB expression, while increased TRAF3 expression in H_2_O_2_-treated hepatocytes, which was normalized by further SEV treatment. Moreover, in the presence of SEV, miR-124-3p inhibitor led to decreased miR-124-3p and CREB expression, whereas increased TRAF3 expression in H_2_O_2_-treated hepatocytes (Fig. 5A). In addition, in the presence of SEV, miR-124-3p mimic treatment elevated the CREB expression in H_2_O_2_-treated hepatocytes (Supplementary Fig. 2B). Treatment of SEV + miR-124-3p inhibitor + oe-CREB led to elevated CREB expression in H_2_O_2_-treated hepatocytes compared with treatment with SEV + miR-124-3p inhibitor + oe-NC (Fig. 5A).

In addition, ELISA depicted that ALT, AST, and GST levels were increased by SEV + miR-124-3p inhibitor in H_2_O_2_-treated hepatocytes, whereas oe-CREB declined the said levels in H_2_O_2_-treated hepatocytes in the presence of SEV + miR-124-3p inhibitor (Fig. 5B). Flow cytometric data demonstrated that hepatocyte apoptosis was notably increased upon SEV + miR-124-3p inhibitor in H_2_O_2_-treated hepatocyte, which was reversed by SEV + miR-124-3p inhibitor + oe-CREB (Fig. 5C). In addition, ELISA and RT-qPCR documented that TNF-α, IL-1β, and IL-6 levels were all markedly elevated upon SEV + miR-124-3p inhibitor treatment in H_2_O_2_-treated hepatocytes, which was then abrogated in the presence of SEV + miR-124-3p inhibitor + oe-CREB (Fig. 5D, E). Subsequently, Western blot analysis results showed that in presence of H_2_O_2_ treatment, SEV + miR-124-3p inhibitor contributed to increased expression of cleaved-caspase-3 and Bax and decreased expression of Bcl-2, while opposite trends were noted in H_2_O_2_-treated hepatocytes treated with SEV + miR-124-3p inhibitor + oe-CREB (Fig. 5F and Supplementary Fig. 1I).

### SEV manipulates miR-124-3p/TRAF3/CREB axis to alleviate the hepatic I/R injury in mice

**Fig. 6:**
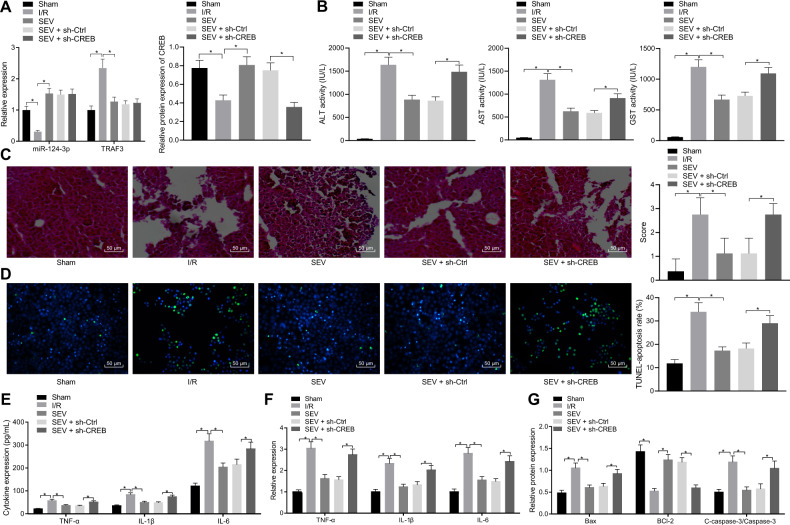
SEV inhibits hepatic I/R injury in mice through the miR-124-3p/TRAF3/CREB axis. **A** The expression of miR-124-3p and TRAF3 mRNA determined by RT-qPCR and CREB protein expression measured by Western blot analysis in liver tissues of sham-operated mice, I/R mice or I/R mice treated with SEV/SEV + sh-CREB. **B** Serum levels of GST, ALT, and AST in sham-operated mice, I/R mice or I/R mice treated with SEV/SEV + sh-CREB detected by ELISA. **C** Liver tissue injury in sham-operated mice, I/R mice or I/R mice treated with SEV/SEV + sh-CREB determined by HE staining (200×). **D** Hepatocyte apoptosis in sham-operated mice, I/R mice or I/R mice treated with SEV/SEV + sh-CREB detected by TUNEL staining (200×). **E** The expression of serum TNF-α, IL-1β, and IL-6 in sham-operated mice, I/R mice or I/R mice treated with SEV/SEV + sh-CREB detected by ELISA. **F** The expression of TNF-α, IL-1β, and IL-6 in liver tissues of sham-operated mice, I/R mice or I/R mice treated with SEV/SEV + sh-CREB detected by RT-qPCR. **G** The protein expression of Bax, Bcl-2, and cleaved-caspase-3 in liver tissues of sham-operated mice, I/R mice or I/R mice treated with SEV/SEV + sh-CREB detected by Western blot analysis. *p < 0.05, *n* = 8.

It was found that compared with sham-operated mice, miR-124-3p and CREB expressions were significantly diminished in liver tissues of I/R mice in addition to increased expression of TRAF3, which was reversed by SEV treatment. SEV + sh-CREB enhanced CREB expression in contrast to SEV + sh-control (Fig. 6A). ELISA data displayed that ALT, AST, and GST levels were dramatically higher in I/R mice than in sham-operated mice, which was reduced upon SEV. Relative to SEV + sh-control treatment, the ALT, AST, and GST levels were elevated upon SEV + sh-CREB treatment (Fig. 6B). HE staining and TUNEL staining further illustrated that compared with the sham-operated mice, I/R mice exhibited enhanced liver tissue injury and apoptosis, while SEV alleviated the said liver tissue injury and inhibited hepatocyte apoptosis caused by I/R modeling. However, SEV + sh-CREB counteracted the changes brought about by SEV (Fig. 6C, D).

Meanwhile, ELISA and RT-qPCR showed that TNF-α, IL-1β, and IL-6 levels in the serum and liver tissues of mice were notably increased following I/R but reversed by SEV treatment. However, the effects caused by SEV were normalized by SEV + sh-CREB (Fig. 6E, F). Western blot analysis results revealed that I/R modeled mice showed increased cleaved-caspase-3 and Bax expression, accompanied by downregulated Bcl-2, whereas SEV brought about the opposite findings in I/R mice, which was neutralized by SEV + sh-CREB (Fig. 6G and Supplementary Fig. 1J).

## Discussion

I/R injury is one of the leading causes of liver damage occurring during hepatic resection and transplantation, significantly contributing to liver dysfunction and post-transplantation failure [18]. Prior evidence has indicated that the prevention of SEV preconditioning against hepatic I/R injury was closely associated with miRNAs along with their downstream signaling axis [8]. Therefore, in this study, our data clarified that miR-124-3p confers hepatoprotective effects against hepatic I/R injury via the TRAF3/CREB axis.

Firstly, our findings revealed that SEV preconditioning rescued the down-regulation of I/R-induced miR-124-3p in mouse and cell models. Furthermore, the I/R-induced liver damage, in addition to serum levels of GST, ALT and AST, hepatocyte apoptosis and inflammation were all relieved by SEV. Hepatic I/R is also accompanied by acute self-sustaining inflammation resulting in necrosis [19]. Similarly, various studies have documented the critical importance of aberrantly expressed miRNAs in hepatic I/R injury, with evidence even dictating alteration of miRNA expression as a promising therapeutic strategy to prevent or alleviate hepatic I/R injury [20]. For instance, upregulation of several miRNAs, such as miR-27a [21] and miR-191 [22] in response to hepatic I/R has been reported. Meanwhile, other miRNAs, such as miR-20a [23] and miR-125b [24] are known to exhibit downregulated levels in hepatic I/R, contributing to diminished inflammation and liver damage by inhibiting the transcription of their target genes. Notably, Li et al. also uncovered that miR-124 was downregulated in I/R animal and cell models, and further suggested that miR-124 could alleviate I/R-triggered liver injury by targeting the Rab38 gene [25]. However, the aforementioned study solely evaluated cell apoptosis levels in response to miR-124 expression alteration, whereas in the current study, several factors such as the levels of liver damage, ALT, AST and GST contents, hepatocyte apoptosis and inflammation were extensively studied using in vivo models. As a result, we hypothesized that both SEV and miR-124 restoration may result in amelioration of hepatic I/R injury.

Subsequently, we explored the role of miR-124 in the alleviation of SEV in hepatic I/R injury in vitro, and came across results indicating that SEV inhibited hepatic I/R injury by elevating the miR-124-3p expression, as evidenced by repressed ALT, AST and GST contents, and diminished hepatocyte apoptosis and inflammation. SEV has been revealed to prevent hepatic I/R injury by reducing ALT/AST contents and histological changes in liver tissues [26]. Moreover, SEV is known to exert its hepatoprotective role on the liver against I/R via different mechanisms, including inflammatory signaling and immunological mechanisms [27]. Additionally, a prior research revealed that SEV possesses the ability to regulate macrophages to confer a protective role during the course of cerebral ischemic injury [28], whereas the effects of SEV on macrophages in hepatic I/R injury remain to be unclear and will be addressed in our future studies. Nevertheless, the miRNA-mediated translational repression of mRNAs has been extensively studied in inflammatory regulation and organ injury following I/R. One particular study highlighted that SEV preconditioning has the potential to suppress oxidative stress and hepatocyte apoptosis caused by I/R modeling through the modulation of miR-200c [29]. Prior evidence has also indicated that miR-124-3p expression was enhanced in SEV treated cell lines, while the restoration of miR-124-3p has been shown to augment the beneficial pharmacological effects of inhaled SEV [12]. Accordingly, we speculated that the promotive effect of miR-124-3p on SEV impact could be achieved through the mediation of target genes.

Consequently, we employed miR target detection program for predicting the potential target genes of miR-124-3p, wherein the TRAF3 gene was predicted as a core candidate target of miR-124-3p, and further verified using a dual-luciferase reporter assay. TRAF3 is a ubiquitously expressed adapter protein, which could accelerate the apoptotic potential of mature B cells. TRAF3 also plays a critical role in inflammation regulated by T cells [30]. In addition, studies have found that ablation of TRAF3 could abrogate hepatocyte death, inflammation and pro-inflammatory cytokine secretion in animal and cell models of hepatic I/R [31]. On the contrary, the adverse role of TRAF3 in liver steatosis has also been unraveled via stimulation of TAK1 ubiquitination and nullification of protein stability [14]. Furthermore, Chen et al. demonstrated that TRAF3 brought about significant elevations in metabolic inflammation and metabolic disease progression in hepatic steatosis, while TRAF3 inhibition was associated with reduced levels of inflammation [32]. Consistently, our experimental findings suggested that overexpression of miR-124-3p relieved the extent of hepatic I/R injury by targeting TRAF3, corresponding to repressed GST, ALT, and AST levels, hepatocyte apoptosis and inflammation. Additionally, mechanistic investigations in the current study illustrated negative-regulation of CREB expression by TRAF3 through ubiquitination, resulting in accelerated progression of hepatic I/R injury. Depleted levels of TRAF3 have also been associated with elevated CREB protein expressions and reduced CREB ubiquitination [15]. Besides, studies have identified the attenuation of CREB activity in an I/R-injured brain, whereas restoration of CREB conferred the opposite effects and relieved I/R injury [16]. The modulation of miR-124 on CREB expression has been demonstrated in constraining synaptic plasticity by a study conducted by Rajasethupathy et al. [33]. Overall, the results of in vitro and in vivo assays in the current study validated that SEV impedes I/R-induced liver damage, GST, ALT, and AST levels, hepatocyte apoptosis and inflammation via mediation of the miR-124-3p/TRAF3/CREB axis.

**Fig. 7:**
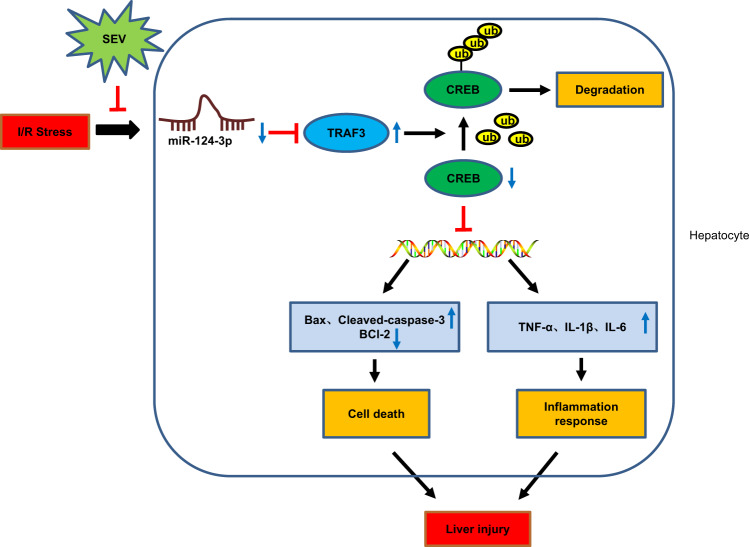
The proposed regulatory mechanism illustrating the role of miR-124-3p, TRAF3, and CREB in hepatic I/R injury. The expression of miR-124-3p was downregulated when hepatic I/R occurs, which resulted in increased expression of TRAF3 targeted by miR-124-3p, promoting the ubiquitination and degradation of CREB. Upregulation of Bax, cleaved-caspase-3, TNF-α, IL-1β and IL-6 expression, as well as promoted hepatocyte apoptosis and inflammatory response further contributes to hepatic I/R injury. SEV promotes the expression of miR-124-3p and resulted in ameliorated hepatic I/R injury.

In conclusion, the current study provides new insight into the hepatoprotective mechanisms of SEV in hepatic IR injury via miR-124-3p. The augmented expression of miR-124-3p regulated by SEV resulted in attenuated hepatic IR injury by impairing TRAF3-mediated CREB ubiquitination. Directly targeting inflammation and hepatocyte apoptosis by manipulating miR-124-3p should be considered as a novel means to combat IR-induced injury in liver surgeries (Fig. 7). However, how does the outcome of this study contributed to therapy/treatment of liver injury needs to be further explored in future studies.

## Materials and methods

### Ethics statement

The current study was conducted under the approval of the Medical Ethics Committee of The First Hospital of China Medical University. Animal experiments were performed following the Guide for the Care and Use of Laboratory Animals published by the US National Institutes of Health, and all efforts were made to minimize the suffering of the included animals.

### Establishment of hepatic I/R injury mouse models

In total, 45 male C57BL/6 mice (aged 8–10 weeks, weighing 18–21 g) purchased from Animal Experiment Center of The First Hospital of China Medical University were enrolled to establish the animal models of hepatic I/R injury. The portal vein and hepatic artery of the middle lobe and left lobe of the liver were clamped for 90 min using a special clamp after the mice were anesthetized with 2% pentobarbital sodium. After 24 h of reperfusion, the mice were subsequently euthanized, and serum and liver specimens were collected for subsequent experimentation. Sham-operated mice (*n* = 8) were only subjected to laparotomy without vascular occlusion or reperfusion, while the I/R mice (*n* = 37) were injected with 2.0% SEV (0426, Fuso Pharmaceutical Industries, Ltd., Osaka, Japan) three times every 15 min in a SEV evaporator (Draeger Medical, Lubeck, Germany) prior to hepatic I/R. Following the instructions of AAV construction, the mouse livers post-anesthesia were administrated with AAV sh-control and sh-CREB. Twenty-four hours after injection, hepatic I/R was performed on the mice. The animal model of hepatic I/R was successfully established in 32 mice, with a calculated success rate of 86.49%. The 32 mice were subsequently assigned into four groups, with eight mice in each group.

### RT-qPCR

mRNA or miRNA was collected from tissues or cells, followed by extraction of tissue RNA using the TRIzol reagent (16096020 or AM1561, Thermo Fisher Scientific Inc., Waltham, MA, USA). For miR-124-3p detection, the total RNA was reversely transcribed using miRNA First Strand cDNA Synthesis (Tailing Reaction) Kit (B532451-0010, Shanghai Sangon Biotechnology Co., Ltd., Shanghai, China). For mRNA detection, 5 μg of tissue RNA was transformed to complementary DNA (cDNA) via reverse transcription according to the manufacturer’s instructions of the RT-qPCR kit (ABI Company, Oyster Bay, NY, USA). U6 functioned as the internal reference for miR-124-3p, while glyceraldehyde-3-phosphate dehydrogenase (GAPDH) served as the reference for the other genes. The fold changes in gene expression were calculated using means of relative quantification (the 2^−^^ΔΔCt^ method). The primers are depicted in Supplementary Table 1.

### Western blot analysis

Cells were rinsed with phosphate buffer saline (PBS), and then lysed with cell lysis buffer (P0013, Beyotime Biotechnology Co., Shanghai, China). The tissues were ground in liquid nitrogen and lysed with cell lysis buffer (P0013, Beyotime), followed by incubation at 4 °C for 30 min. After incubation, the lysate was collected into a 1.5 ml Eppendorf tube, and then centrifuged at 4 °C and 12,000 × *g* for 15 min, and the supernatant was collected. Sample protein concentration was determined using a bicinchoninic acid (BCA) protein detection kit (Beyotime). The protein loading buffer was added to the supernatant and boiled for 5 min. Subsequently, 20 μg of protein was resolved in 10% sodium dodecyl sulfate-polyacrylamide gel electrophoresis and placed onto a polyvinylidene fluoride membrane (Millipore, Billerica, MA, USA). Following blockade with 5% skim milk, the membrane was incubated for one night at 4 °C along with Tris-buffered saline containing Tween-20 (TBST)-diluted primary antibodies (Abcam Inc., Cambridge, UK) to TRAF3 (ab36988, dilution ratio of 1–2 μg/ml), CREB (ab31387, dilution ratio of 1: 1000), cleaved-caspase-3 (ab2302, dilution ratio of 1: 500), Bax (ab32503, dilution ratio of 1: 1000), Bcl-2 (ab182858, dilution ratio of 1: 2000), ubiquitin (Ub; ab7780, dilution ratio of 1: 2000), and β-actin (used as internal reference; ab8267, dilution ratio of 1: 10,000). Following three rinses with TBST, the membrane was then immunoblotted with horseradish peroxidase-labeled secondary antibodies (ab205718, goat anti-rabbit, dilution ratio of 1: 10000; ab205719, goat anti-mouse, dilution ratio of 1: 10,000), followed by 1-hour incubation at room temperature. The blots were developed utilizing enhanced chemiluminescence reagent (Baoman Biotechnology, Shanghai, China) and band areas were quantified using the gel image analysis software, Image J Software.

### ELISA

An ADVIA 2400 biochemical analysis instrument (Siemens, Tarrytown, NY, USA) was employed to detect the serum levels of ALT, AST, and GST. The levels of ALT, AST, GST, TNF-α, IL-1β, and IL-6 in the supernatant were detected using an ELISA kit (PeproTech, Rocky Hill, NJ, USA) following the kit instructions.

### HE staining

Paraffin-embedded mouse livers were sectioned, and successively immersed in xylene I and II (each for 10 min), in anhydrous ethanol I and II (each for 5 min) and in gradient alcohol (95, 90, 80, and 70%, each for 5 min). Following a rinse with distilled water, the sections were dewaxed, dehydrated, and subsequently soaked in Harris hematoxylin for 3–8 min. Following differentiation in 1% hydrochloric acid alcohol for several seconds, the sections were immersed in 0.6% ammonia to return to blue coloration. Subsequently, the sections were stained with eosin for 1–3 min, followed by treatment with gradient alcohol (95%, 5 min) and anhydrous ethanol I and II (each for 5 min). Next, the sections were dehydrated and cleared in xylene I and II (each for 5 min). The sections were then removed from xylene, naturally dried, and sealed with neutral gum. Finally, the sections were observed under a microscope, and images were captured for further analysis. All samples were analyzed using the blind method. The grade of liver injury was evaluated using the Suzuki’s criterion on a scale from 0–4 [34], which indicated the severity of sinusoidal congestion, cytoplasmic vacuolation, and parenchymal cell necrosis (Supplementary Table 2).

### TUNEL staining

The paraffin sections of mouse livers were immersed in gradient ethanol (100, 95, 90, 80, and 70%, 3 min each) following two rinses in xylene (5 min each). The sections were then treated with proteinase K working solution (20 μg/ml) at room temperature for 15–30 min, and then reacted with PBS containing 2% hydrogen peroxide at room temperature for 5 min. After preparation of the TUNEL reaction mixture, the treatment group was mixed with 50 μl TdT and 450 μl fluorescein-labeled dUTP solution, while the NC group was added with 50 μl fluorescein-labeled dUTP solution. The positive control group was firstly added with 100 μl DNase 1, and then incubated for 10–30 min at room temperature, while the subsequent steps were similar as performed in the treatment group. For the treatment group, the sections were incubated with 50 μl TUNEL reaction mixture in a dark wet box with humidity at room temperature for 1 h, while 50 μl of the fluorescein-labeled dUTP solution was supplemented to the NC group. Apoptotic cells were counted under a fluorescence microscope (excitation light wavelength: 450–500 nm; detection wavelength: 515–565 nm). The sections were subsequently treated with 50 μl DIG-POD at room temperature for 30 min, and with 50–100 μl diaminobenzidine substrate for 10 min. Next, hematoxylin or methyl green was employed to counterstain the sections once photographed. The sections underwent dehydration with gradient alcohol, clearing with xylene and sealing with neutral gum. Finally, the apoptotic cells (200–500 cells in total) were observed under a light microscope and the morphology was photographed. Apoptotic cells were then comprehensively judged based upon morphological characteristics. The unstained cells presented with a smaller size, and complete but foaming cell membrane; whereas the apoptotic bodies appeared in the late stage, and the adherent cells were shrunk and became round, and then fall off; while the stained cells showed concentrated and marginalized chromatin into massive/apoptotic bodies, along with cracked nuclear membrane.

### Flow cytometry

Cells in the culture dish were collected after being detached with ethylenediaminetetraacetic acid-free trypsin. The cells in the supernatant were then centrifuged at 2000 rpm for 5 min and collected. The collected cells were stained with 5 μl fluorescein isothiocyanate (FITC)-labeled Annexin V (Annexin V-FITC) and 5 μl propidium iodide (KGA106, Nanjing Keygen Biotech CO., LTD, Nanjing, China) for 15 min. Cell apoptosis was detected using a flow cytometer (BD Biosciences, Franklin Lakes, NJ, USA).

### Cell treatment

Normal BNL CL.2 mouse hepatocytes (Shanghai Institute for Biological Sciences, Chinese Academy of Sciences, Shanghai, China) were cultured in Dulbecco’s modified Eagle’s medium containing 10% fetal bovine serum, 100 μg/ml streptomycin, and 100 U/ml penicillin (Gibco, Carlsbad, California, USA) at 37 °C with 5% CO_2_ and 95% O_2_. The hepatocytes were subsequently detached with 0.25% trypsin and sub-cultured at a ratio of 1:3. When the cells were at the logarithmic phase of growth, the cells were seeded in a six-well plate at a density of 3 × 10^5^ cells/well. Upon reaching 70–80% confluence, the hepatocytes were transfected according to the manufacturer’s instructions of Lipofectamine 3000 reagent (L3000008, Invitrogen, Carlsbad, California, USA). H_2_O_2_-induced oxidative stress model: hepatocytes at 70% confluence were treated with H_2_O_2_ at varying concentrations of 0, 50, 100, 150, 200, 250, and 300 μM for 6 h and then collected. miR-124 expression was measured by RT-qPCR as described above.

Hepatocytes not receiving any treatment were regarded as the control. Hepatocytes were treated with H_2_O_2_ for 5 h to induce I/R model (H_2_O_2_ group), or intervened with SEV for 2 h and then treated with H_2_O_2_ for 5 h to induce I/R model (SEV group). Afterwards, hepatocytes were transfected with NC inhibitor plasmid (SEV + NC inhibitor group), miR-124-3p inhibitor plasmid (SEV + miR-124-3p inhibitor group), miR-124-3p inhibitor + oe-NC plasmids (SEV + miR-124-3p inhibitor + oe-NC group), or miR-124-3p inhibitor + oe-CREB plasmids (SEV + miR-124-3p inhibitor + oe-CREB group) prior to SEV intervention and H_2_O_2_ modeling. Also, hepatocytes were transfected with NC mimic + oe-NC plasmids (NC mimic + oe-NC group), miR-124-3p mimic + oe-NC plasmids (miR-124-3p mimic + oe-NC group), oe-TRAF3 plasmid + NC mimic (oe-TRAF3 + NC mimic group), miR-124-3p mimic + oe-TRAF plasmids (miR-124-3p mimic + oe-TRAF3 group), oe-NC plasmid (oe-NC group), oe-TRAF3 plasmids (oe-TRAF3 group), or oe-TRAF3 and oe-CREB plasmids (oe-TRAF3 + oe-CREB group), and then treated with H_2_O_2_ to induce I/R model. Also, hepatocytes were only transfected with oe-NC plasmid, oe-TRAF3 plasmid, or oe-TRAF3 + oe-CREB plasmid. All the transfection sequences and plasmids used were purchased from GenePharma Company (Shanghai, China). After 48 h of transfection, the hepatocytes were collected for subsequent experimentation. The I/R cell model was established by treating hepatocytes with H_2_O_2_ (5 mM) for 5 h, whereas for SEV treatment, 2% SEV was used to intervene the hepatocytes for 2 h.

### IP assay

In order to detect the ubiquitination levels of CREB, Flag-TRAF3, Myc-CREB, and HA-Ub plasmids were co-transfected into BNL CL.2 cells. Following 48 h of transfection, the total protein was extracted from the cells and quantified using the BCA method. Afterwards, 1 mg total protein was incubated with 30 μl Protein G Agarose and 1 μg normal rabbit immunoglobulin G/primary antibody. Centrifugation was then carried out to remove the supernatant, followed by denaturation of precipitates. The protein was subsequently transferred onto a polyvinylidene fluoride membrane after agarose-gel electrophoresis separation. Blots were then probed with primary antibodies to Flag [#14793, dilution ratio of 1: 5000, rabbit antibody, Cell Signaling Technologies (CST), Beverly, MA, USA], Mcy (ab32072, dilution ratio of 5 µg/ml, rabbit antibody, Abcam), and HA (ab1424, dilution ratio of 10 µg/ml, mouse antibody, Abcam), followed by 1-h incubation at 4 °C. Afterwards, the bands were detected using an enhanced chemiluminescence reagent (Baoman Biotechnology, Shanghai, China), and the gray value for each band was analyzed using the gel image analysis software, Image J Software [35–38].

### Protein stability test

In order to test the effect of ubiquitination on CREB protein stability, 48 h post-transfection with TRAF3 and empty plasmids, cells were incubated with CHX (10 μg/ml, HY-12320, MCE) at 37 °C. The cells were harvested at the 0-, 1-, 2-, 4-, and 8-h time intervals. The protein expression patterns of CREB were quantified using Western blot analysis. The degradation curve of CREB was drawn subsequently.

### Dual-luciferase reporter gene assay

The target gene of miR-124-3p was predicted using a miRNA bioinformatics software. The TRAF3 3'untranslated region gene fragment was artificially synthesized and introduced into the reporter plasmid pmiR-reporter (Huayueyang Biotechnology Co., Ltd., Beijing, China) with the help of endonuclease sites, *Spe I* and *Hind III*. The complementary sequence mutation site of seed sequence was designed based on the TRAF3 WT. T4 DNA ligase was then used to insert the target fragment into the pmiR-reporter plasmid following restriction endonuclease digestion. The luciferase reporter plasmids WT and MUT were co-transfected with miR-124-3p into BNL CL.2 cells. The cells were collected 48 h after transfection, and the protein was extracted. Luciferase activity was tested using a luciferase detection kit (K801-200, BioVision, Palo Alto, USA) with a lomax20/20 luminometer fluorescence detector (Promega Corporation, Madison, WI, USA).

### Statistical analysis

Data analyses were performed using SPSS 21.0 software (IBM Corp., Armonk, NY, USA). Measurement data were presented as mean ± standard deviation. Paired data between two groups obeying normal distribution and homogeneous variance were compared utilizing paired *t*-test. Data among multiple groups were compared using one-way analysis of variance (ANOVA) and a Tukey’s test. Data among groups at different time points were compared by repeated-measures ANOVA and Bonferroni post hoc test. A value of *p* < 0.05 reflected a statistically significant difference.

## Supplementary information

Supplementary materials

## Data Availability

The original contributions presented in the study are included in the article/Supplementary Material, further inquiries can be directed to the corresponding author.
