# Supplementary material for: Hepatoprotective effects of sevoflurane against hepatic ischemia-reperfusion injury by regulating microRNA-124-3p-mediated TRAF3/CREB axis
Source: Cell Death Discov. 2022 Mar 8;8:105. doi: 10.1038/s41420-021-00784-7 (PMC8904859; doi:10.1038/s41420-021-00784-7)
Supplement: Supplementary file 1 — Supplementary materials [file 41420_2021_784_MOESM1_ESM.docx]

**
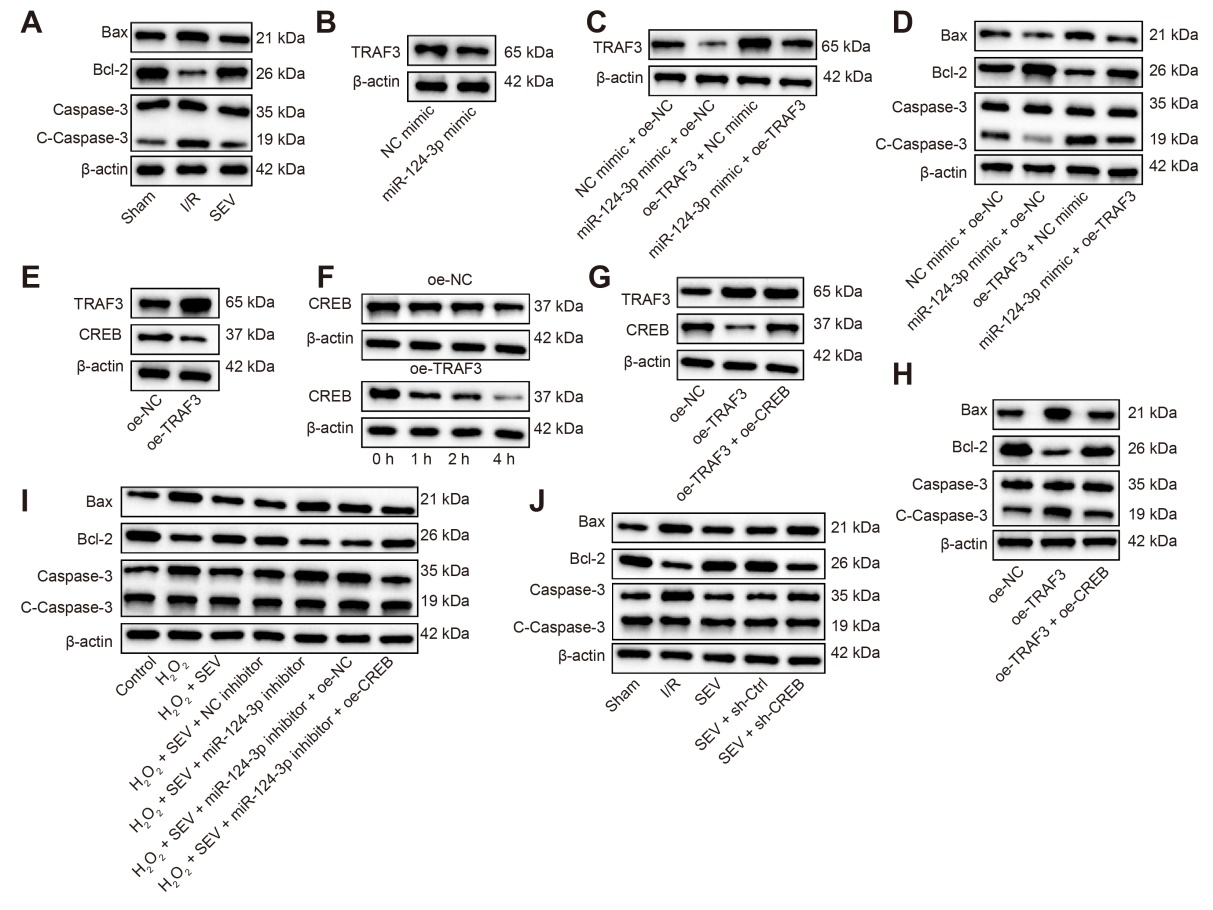
**

**Supplementary Figure 1.** Original western blots. A, The protein expression of Bax, Bcl-2, and cleaved-caspase-3 in liver tissues of sham-operated mice, I/R mice or I/R mice treated with SEV detected by Western blot analysis. B, The expression of TRAF3 in the presence of miR-124-3p mimic detected by Western blot analysis. C, The expression of miR-124-3p and TRAF3 in hepatocytes in the presence of miR-124-3p mimic, oe-TRAF3 or miR-124-3p mimic + oe-TRAF3 detected by Western blot analysis. D, The protein expression of Bax, Bcl-2, and cleaved-caspase-3 in hepatocytes in the presence of miR-124-3p mimic, oe-TRAF3 or miR-124-3p mimic + oe-TRAF3 detected by Western blot analysis. E, The protein expression of TRAF3 and CREB in the presence of oe-TRAF3 or oe-TRAF3 + oe-CREB detected by Western blot analysis. E, The effects of TRAF3 on CREB protein stability after CHX intervention. G, The expression of CREB and TRAF3 in hepatocytes in the presence of oe-TRAF3 or oe-TRAF3 + oe-CREB detected by Western blot analysis. H, The protein expression of Bax, Bcl-2, and cleaved-caspase-3 in hepatocytes in the presence of oe-TRAF3 or oe-TRAF3 + oe-CREB detected by Western blot analysis. I, The protein expression of Bax, Bcl-2, and cleaved-caspase-3 in hepatocytes in the presence of SEV, SEV + miR-124-3p inhibitor or SEV + miR-124-3p inhibitor + oe-CREB detected by Western blot analysis. J, The protein expression of Bax, Bcl-2, and cleaved-caspase-3 in liver tissues of sham-operated mice, I/R mice or I/R mice treated with SEV/SEV + sh-CREB detected by Western blot analysis.

**
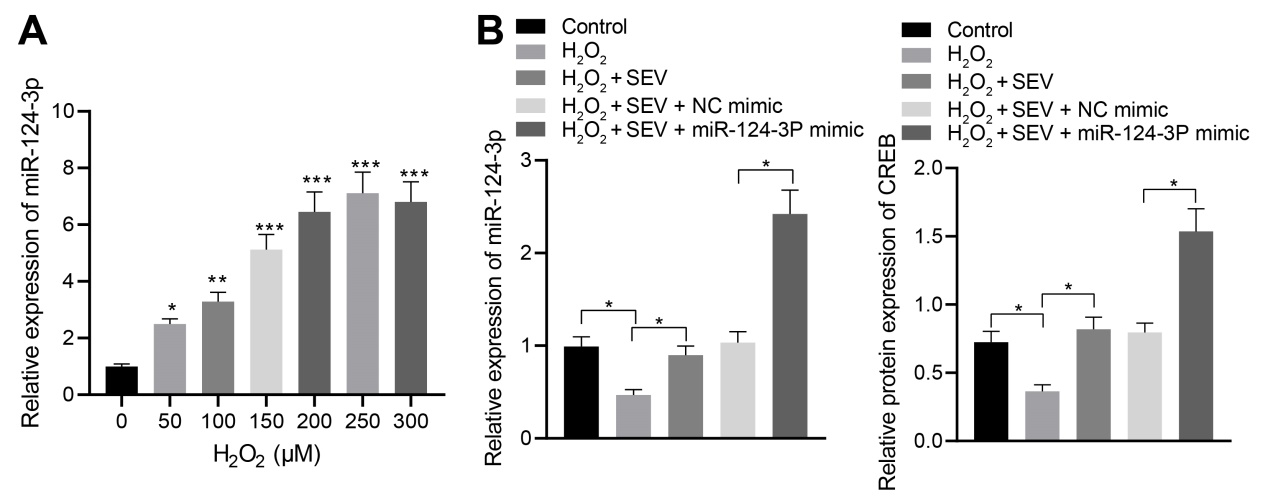
**

**Supplementary Figure 2.** miR-124-3p expression and CREB protein expression after different treatment. A, The expression of miR-124-3p after treatment with different concentrations of H_2_O_2_. * *p* < 0.05, ** *p* < 0.01, *** *p* < 0.001 *vs.* treatment with 0 μM H_2_O_2_. B, miR-124-3p expression detected by RT-qPCR and the expression of CREB protein detected by Western blot analysis after treatment with miR-124-3p mimic. * *p* < 0.05.

**Supplementary Table 1** Primer sequences for RT-qPCR

| Gene | Primer sequences (5’-3’) |
| --- | --- |
| miR-124-3p | F: 5'-GGGTAAGGCACGCGGT-3' |
|  | Universal reverse primer |
| TRAF3 | F: 5'-TGAAGGATAAGGCTGGCACG-3' |
|  | R: 5'-TTTTCTTTGCAGTCGGCACG-3' |
| CREB | F: 5'-GAGAAGCGGAGTGTTGGTGA-3' |
|  | R: 5'-ACTCTGCTGGTTGTCTGCTC-3' |
| TNF-1α | F: 5'-ACCCTCACACTCACAAACCA-3' |
|  | R: 5'-ACCCTGAGCCATAATCCCCT-3' |
| IL-1β | F: 5'-TGCCACCTTTTGACAGTGATG-3' |
|  | R: 5'-TTCTTGTGACCCTGAGCGAC-3' |
| IL-6 | F: 5'-GCCTTCTTGGGACTGATGCT -3' |
|  | R: 5'-TGTGACTCCAGCTTATCTCTTGG-3' |
| U6 | F: 5'-GCTTCGGCAGCACATATACTAAAAT-3' |
|  | Universal reverse primer |
| GAPDH | F: 5'-CCAGCTACTCGCGGCTTTA-3' |
|  | R: 5'-TTCCCATTCTCGGCCTTGAC-3' |

Note: RT-qPCR, reverse-transcription quantitative polymerase chain reaction; miR-, microRNA-; TRAF3, TNF receptor-associated factor 3; CREB, cAMP-responsive element-binding protein 3; TNF-α, tumor necrosis factor-α, IL-, interleukin-; GAPDH, glyceraldehyde-3-phosphate dehydrogenase; F, forward; R, reverse.

**Supplementary Table 2.** Histological criteria for the assessment of liver damage after severe ischemia and reperfusion injury

| Numerical assessment | Congestion | Vacuolization | Necrosis |
| --- | --- | --- | --- |
| 0 | None | None | None |
| 1 | Minimal | Minimal | Single-cell necrosis |
| 2 | Mild | Mild | -30% |
| 3 | Moderate | Moderate | -60% |
| 4 | Severe | Severe | > 60% |
